# The relationship between dietary patterns and depression mediated by serum levels of Folate and vitamin B12

**DOI:** 10.1186/s12888-020-2455-2

**Published:** 2020-02-13

**Authors:** Maryam Khosravi, Gity Sotoudeh, Maryam Amini, Firoozeh Raisi, Anahita Mansoori, Mahdieh Hosseinzadeh

**Affiliations:** 1 Department of Nutrition, School of Medicine, Mashhad University of medical Sciences, Mashhad, Iran; 2 Department of Public Health, North Khorasan University of medical Sciences, Bojnurd, Iran; 3 Department of Community Nutrition, School of Nutritional Sciences and Dietetics, Tehran University of Medical Sciences, Hojatdoost Street, Naderi Street, Keshavarz Blvd., Tehran, Iran; 4 Department of Nutrition Research, Faculty of Nutrition Sciences and Food Technology, National Nutrition and Food Technology Research Institute, Shahid Beheshti University of Medical Sciences, No. 7., Hafezi St., Farahzadi Blvd., Qods Town, 19395-4741, Tehran, 1981619573 Iran; 5 Department of Psychiatry, Roozbeh Hospital and Psychiatry and Psychology Research Centre, Tehran University of Medical Sciences, Tehran, Iran; 6 Nutrition and Metabolic Diseases Research Center, Ahvaz Junishapur University of Medical Sciences, Ahvaz, Iran; 7 Department of Nutrition, Shahid Sadoughi University of Medical Sciences, Yazd, Iran

**Keywords:** Depression, Epidemiology, Dietary pattern, Mediation analysis, Total Homocysteine, Tryptophan, Folate, Vitamin B12

## Abstract

**Background:**

Major depressive disorder is among main worldwide causes of disability. The low medication compliance rates in depressed patients as well as the high recurrence rate of the disease can bring up the nutrition-related factors as a potential preventive or treatment agent for depression. The aim of this study was to investigate the association between dietary patterns and depression via the intermediary role of the serum folate and vitamin B12, total homocysteine, tryptophan, and tryptophan/competing amino acids ratio.

**Methods:**

This was an individually matched case-control study in which 110 patients with depression and 220 healthy individuals, who completed a semi-quantitative food frequency questionnaire were recruited. We selected the depressed patients from three districts in Tehran through non-probable convenience sampling from which healthy individuals were selected, as well. The samples selection and data collection were performed during October 2012 to June 2013. In addition, to measure the serum biomarkers 43 patients with depression and 43 healthy people were randomly selected from the study population. To diagnose depression the criteria of Diagnostic and Statistical Manual of Mental Disorders, fourth edition, were utilized.

**Results:**

The findings suggest that the healthy dietary pattern was significantly associated with a reduced odds of depression (OR: 0.75; 95% CI: 0.61–0.93) whereas the unhealthy dietary pattern increased it (OR: 1.382, CI: 1.116–1.71). The mediation analysis showed that the healthy dietary pattern was associated with a reduced risk of depression via increased serum levels of the folate and vitamin B12; however, the unhealthy dietary pattern was associated with increased risk of depression via decreased serum levels of folate and vitamin B12, based on tree adjusted logistic regression models.

**Conclusion:**

Dietary patterns may be associated with depression by changing the serum levels of folate and vitamin B12. Further studies are required to confirm the mechanism.

## Background

Depression is considered as one of the most common psychological conditions. It is a major causative factor for the disability-adjusted life years lost in the world and currently 322 million people in the world are suffering from the disorder [1]. Depression is highly prevalent in the developed and developing countries. Nearly 16 million American adults have experienced a major depression, in 2015 [2]. In Iran, results of investigations show that 4.1% of adults are affected by major depressive disorders (MDD), while women are 1.95 times more probable to develop MDD than men [3].

It has been long confirmed that diet affects depression. Previous studies investigating the association between diet and depression have mostly considered on food groups [4, 5], foods [6] or nutrients [7]; whereas, few studies have focused on the dietary patterns. Considering the complex interactions between nutrients and foods, dietary pattern approach was suggested by nutritional epidemiologists to study the diet-disease relationships [8]. Morever, this approach provides a more comprehensive insight into the diet-brain relation [9].

There is a body of documents indicating the association between dietary patterns and risk of depression. A study on Australian women showed that lower odds of depression was associated with a “traditional” dietary pattern including high amounts of vegetables, fruit, meat, fish, and whole grains [9]. Another study suggested adherence to “processed food” as a dietary pattern increased the risk of depression, while “whole food” consumption decreased it among British middle-aged women. The finding was confirmed by other researchers [10, 11]. There are some studies which have considered the potential role of biomarkers and food intake in depression simultaneously [12, 13].

The relationship between depression and dietary intakes of B6 [14], folate, and B12 [7, 15] have been reported. Moreover, total homocysteine (tHcy), tryptophan (Trp), and tryptophan/competing amino acids (Trp/CAA) ratio were found to be associated with depression [16, 17].

However, the mechanism(s) by which dietary factor(s) affect depression is not clearly understood. Previous studies, on the association between food patterns and depression rarely considered biochemical measures along with dietary patterns.

Ergo, the mechanism through which dietary intake patterns and depression are co-related is not clearly understood. In the present study, the mediatory role of folate, vitamin B12, tHcy, Trp, and Trp/CAA ratio in modifications of dietary patterns to decrease risk of depression is investigated.

## Methods

### Sample size and study design

It was an individually matched case-control observational study. To extract dietary patterns, 110 patients with depression and 220 healthy individuals were recruited. The participants included 260 female (87 depressed and 173 healthy) and 70 male (22 depressed and 48 healthy). We selected the depressed patients from psychiatric clinic of Baharloo hospital in district 15, Imam Hossein hospital in district 7 and Tehran University student counseling center in district 6 through non-probable convenience sampling. After adjusting for age and sex, healthy individuals were selected from the area of residence of hospital patients and students of Tehran University. The samples selection and data collection were performed during October 2012 to June 2013.

To measure serum folate, vitamin B12, tHcy, Trp, and Trp/CAA ratio a total of 86 individuals (*n* = 43 equally distributed in case and control groups) were randomly selected from the participants.

### Inclusion criteria

People with an age range of 18 to 65 years, resided in Tehran, and who have been diagnosed with the disease for a maximum of 3 months were entered in case group. The participants included 260 female (87 depressed and 173 healthy) and 70 male (22 depressed and 48 healthy).

### Exclusion criteria

People with cognitive impairment or other psychotic illnesses, severe depression or lacking the ability to cooperate and answer the questions, hormonal disorders such as Addison’s, Cushing’s disease, hyperthyroidism, hypothyroidism, hyperparathyroidism, cancer, heart disease, diabetes, stroke, fibromyalgia, kidney, or liver failure, multiple sclerosis, Parkinson disease, a history of trauma, cuts, fractures, bleeding, burns, and other similar events in the past 3 months resulting in unconsciousness and hospitalization, chronic and infectious diseases such as HIV, mononucleosis, tuberculosis, viral hepatitis, and pneumonia in the past 2 weeks, those who consumed anti-depression drugs, those who had addiction to alcohol or drugs at the time of the study or in the past 3 months, body mass index (BMI) ≥40 kg/m2, pregnancy and lactation at the time of the study or in the past year, a special diet in the past 2 months, any type of special diet for more than 2 months in the past year, B12 injection more than once in the past 6 months, B-complex injection more than once per month for at least 6 months in the last year, and any dietary supplement in injection or oral form in the past 3 months were excluded from the study.

### Recruitment and matching

Our samples were recruited from two psychiatric clinics in Tehran, capital of Iran. The participants’ major depressive disorder(s) was diagnosed by psychiatrists using the criteria of the Diagnostic and Statistical Manual of Mental Disorders, fourth edition (DSM-IV) [18]. These patients had no history of depression in the past year. For the control group, lack of major depression disorder(s) was confirmed based on Beck Depression Inventory questionnaire (BDI-II) [19], standardized in Iran [20]. The control group members had no history of depression in the past year.

To invite eligible individuals, the interviewers went to each patient’s residential area and described the aim of the study. Patients were matched with controls based on gender, age, and residential area. Each patient with depression was matched with two members of the control group.

To identify the dietary patterns, the open Epi software was used [21, 22].

### Assessment of covariates

A demographic questionnaire was used to collect information about participants’ general characteristics and some confounders. To calculate BMI weight and height were obtained from all participants. For quantitative measurement of anxiety, as a confounder, the Iranian standardized Beck Anxiety Inventory was utilized [23]. Participants’ dietary intakes in the last 12 months were assessed using a valid and reliable semi-quantitative food frequency questionnaire (FFQ) [24]. A validated physical activity questionnaire consisting of nine levels of activity was applied [25, 26].

### Assessment of biochemical markers

In order to measure the biochemical markers, 5 ml blood samples were collected from participants after 12 h of fasting. The samples were transferred into tubes with no anticoagulant. After centrifuging for 20 min at 1500 g in room temperature, the serum was separated and stored at − 70 °C. Serum folate and vitamin B12 were measured by gamma method (SimulTRAC-SNB Radioassay Kit; Becton Dickinson, Orangeburg, NY, USA). Total homocysteine (tHcy) concentration was also analyzed using the modified immuno-enzyme method (Axis-Shield, UK). Plasma levels of amino-acids were determined by a modern High-Performance Liquid Chromatography (HPLC) device with fluorescent detector. Initial preparation involved the precipitation of serum protein with methanol. Simultaneous derivatization was performed with ortho-phthaldehyde. After derivatization, 10 μl of each sample was injected into the HPLC device. The device model was YoungLin Acm 3000 HPLC (Young Lin Instrument Co. Ltd., Anyang, Korea). Later, HPLC was carried out on a 4.6 × 250 mm column of Inertsil ODS 5 μm, at 37 °C in the wavelength range of 340–420 nm, which took one hour for each sample. Within- and between-assays precisions were 4.8–6.8 and 6.5–8.5 for all amino acids, respectively.

### Statistical analysis

The normality of covariates was tested by Kolmogorov-smirnov. To compare variables in two groups t-test or Mann-Whitney were used. The association between dietary patterns and depression was investigated by logistic regression models after adjusting for the confounders in multiple logistic regression models. In addition, the goodness of fit for these logistic regression models was examined by logical confidence intervals and Hosmer and Lemeshow test. Furthermore, we used multiple logistic regression to test the mediatory variables [27].

The mediatory analysis was designed to determine the mediator variable, among the biochemical markers, in the relationship between dietary patterns and depression. Logistic regression was employed due to the dichotomous nature of the dependent variable. We designed tree models to determine whether a variable was a mediator or not.

After these analyses, we selected the mediator variables in the case that:

1. A significant association was confirmed between dietary patterns and depression
2. By adding the mediator variables into the first model, the relationship between dietary patterns and depression did not remain significant. Since dietary pattern is related to depression via mediator variables, by adding mediators into the model, the relationship between dietary patterns and depression is transferred into the relationship between mediator variables and depression. Therefore, a third model should be designed to ensure the significant relationship between the mediator variables and depression.
3. In the third model, the relationship between the mediator variables and depression should be significant [27].

The healthy dietary pattern in this study was defined as high in fruits as well as cruciferous, yellow, green, leafy, and other vegetables, low fat dairy, whole grains, nuts, and olives. Unhealthy dietary pattern was high in refined grains and breads, high fat dairy, solid oils, liquid oils and mayonnaise, pickle, snacks, soft drinks, industrial fruits and juice, red meats, poultry, processed meats, and sweets.

In the current study, the mediatory analysis was performed after adjusting for the confounding variables. In other words, the confounding variables were present in all three aforementioned models. All statistical analyses were carried out using SPSS (version 20; Chicago, IL).

## Results

Some comparisons between groups are shown in Tables 1 and 2. Physical activity as a confounder was significantly higher in control group than the patients (*p* = 0.004) and daily energy intake was not significantly different between groups (*p* = 0.06).

**Table 1:** Comparison of quantitative variables between depressed groups (cases) and control groups

Quantitative variables	Controls	Cases	*P*-value
Energy (Kcal) ^b^	2634 ± 69	2887 ± 112	0.06
Years of education^b^	12.2 ± 0.3	11.2 ± 0.46	0.04
Birth rank^b^	2.9 ± 0.12	3.07 ± 0.19	0.4
Family Number^b^	3.7 ± 0.15	3.8 ± 0.08	0.2
Cigarette use (No./week)^b^	8.2 ± 2.8	2.6 ± 1.6	0.09
Hookah use (times/week) ^b^	0.4 ± 0.22	0.08 ± 0.02	0.6
Physical activity (MET-hr/d) ^a^	38.6 ± 0.33	36.9 ± 0.52	0.007
BMI (kg/m^2^) ^a^	26.4 ± 0.49	26.4 ± 0.37	0.9
Anxiety score^a^	7.2 ± 0.43	20.6 ± 0.96	0.001

^a^ Independent samples T test ^b^ Mann-Whitney test *BMI* body mass index

**Table 2:** Comparison of qualitative variables between non-depressed and depressed groups

Qualitative variables	Categories	Control No. (%)	Cases No. (%)	P^a^_value_
Marital status	Single	67 (67.7)	32 (32.3)	0.2
	Married and living with spouse	143 (68.1)	67(31.9)	
	Married and living apart from spouse, divorced or Widow	11 (50.0)	11 (50.0)	
Depression history	No	77 (27.9)	199 (72.1)	<0.001
	Yes	22 (40)	33 (60)	
	Yes	47 (21)	68 (63)	
Job	employees	42 (77.8)	12 (22.2)	0.01
	Housekeeper &retired	104 (60.1)	69 (39.9)	
	free job	32 (66.7)	16 (33.3)	
	students	38 (80.9)	9 (19.1)	
Periods of unemployment in the past 5 years	0	99 (75.6)	32 (24.4)	0.01
	< 6 months	5 (100)	0 (0)	
	≥6 months	19 (54.9)	13 (40.6)	
	house worker	98 (60.1)	65 (39.9)	
Distressing life events in the last 6 months	no	174 (74.4)	60 (25.6)	0.001
	yes	47 (49.5)	48 (50.5)	
Distressing childhood events	no	171 (71.0)	70 (29.0)	0.01
	yes	50 (56.2)	39 (43.8)	
Physical activity (MET-h/day)	Very light	41 (50)	41 (50)	0.004
	Light	56 (68.3)	26 (31.7)	
	medium	59 (73.8)	31 (26.2)	
	Intense	59 (72.8)	22 (27.2)	

^a^ Chi-Square Test MET-h/day: metabolic equivalent hours per day

According to the findings, we recognized two dietary patterns of healthy and unhealthy foods. The healthy dietary pattern led to significantly lower odds ratio for depression (OR: 0.39, CI: 0.17–0.92), whereas, the unhealthy dietary pattern resulted in significantly higher odds ratio (OR: 2.6, CI: 1.04–6.08). Table 3 illustrates the relationship between biochemical markers and depression before and after adjustment for the confounding factors. Based on the information tabulated in Table 3, just folate and vitamin B12 had significant relationship with depression.

**Table 3:** The comparison of the serum biochemical markers between case and control groups

Biochemical factors	Case (Mean ± SE)	Control (Mean ± SE)	*P*_value_^a^	P _value_^b^	OR(95%CI)
Folate (ng/ml)	5.8 ± 0.3	7.7 ± 0.28	<0.001	<0.001	0.54 (0.38–0.75)
Vitamin B12(pg/ml)	550.9 ± 55.1	954.9 ± 57.7	<0.001	<0.001	0.996 (0.993–0.998)
Hcy^1^ (μmol/l)	11.2 ± 1.1	11.9 ± 0.9	0.2	0.5	0.97 (0.9–1.05)
Trp^2^(μmol/l)	70.3 ± 2.9	75.3 ± 3.9	0.3	0.1	0.97 (0.94–1.01)
Tryptophan/competing amino acids^3^	0.113 ± 0.003	0.104 ± 0.002	0.02	0.5	1.46 (0.45–4.76)

^a^ T- test for Square, square root or logarithm

^b^ Multiple logistic regression after adjusting for job, education, marital status, children number, smoking and hookah use, depression history, unemployment history in the past 5 year, tragic events in the past 6 months and the whole lifetime, energy intake, physical activity and dietary patterns

^1^ Homocystein

^2^ Tryptophan

^3^ Valine, leucine, tyrosine, phenylalanine and isoleucine (These amino acids compete for the same cerebral uptake mechanism)

The results showed that dietary patterns and depression were co-related via the intermediary role of folate and vitamin B12, which was initially due to the significant association of dietary patterns with depression as well as the significant association of folate and vitamin B12 with depression. Table 4 illustrates the association between dietary patterns and depression after adjusting for the confounding factors (Model 1). To test the hypothesis, folate and vitamin B12 were added into the regression model 2. By adding these covariates, the significant association between dietary patterns and depression was eliminated. Therefore, these vitamins can be assumed as intermediate variables. To confirm the results, the relation of folate and vitamin B12 with depression was examined (Table 4 - model 3). This mediatory role was confirmed with the significant results of model 3.

**Table 4:** Mediation analysis for the relationship between dietary patterns and depression

dietary patterns	Model	1 p _value_	2 p _value_	3 p _value_	OR(95%CI)
Healthy dietary pattern	Folate	0.028	0.10	< 0.001	0.4 (0.25–0.66)
	Vitamin B12	0.028	0.17	< 0.001	0.995 (0.993–0.998)
Unhealthy dietary pattern	Folate	0.020	0.30	< 0.001	0.5 (0.35–0.72)
	Vitamin B12	0.020	0.60	< 0.001	0.995 (0.993–0.998)

Model 1: Logistic regression model for studying the relation between depression and quartiles of dietary patterns

Model 2: Logistic regression model for studying the relation between quartiles of healthy dietary pattern and depression with mediation variables

Model 3: Logistic regression relationship between mediation variables and depression

All of 3 models were adjusted for job, education, marital status, children number, smoking and hookah use, depression history, unemployment history in the past 5 year, tragic events in the past 6 months and the whole lifetime, energy intake, physical activity and dietary patterns

Based on Table 4, the healthy dietary pattern is related to decreased depression by increasing the serum level of folate and vitamin B12. However, the unhealthy dietary pattern is related to increased depression via reduction of the serum level of folate and vitamin B12. All three models were adjusted for participants’ job, education, marital status, children number, smoking and hookah use, depression history, unemployment history in the past 5 years, tragic events in the past 6 months and through the whole lifetime, daily energy intake, physical activity, and dietary patterns.

## Discussion

We compared a number of variables among the two groups and there was not a significant difference. It shows that we’ve matched enough cases and controls sufficiently.

Of course physical activity as a confounder was significantly higher in controls than the patients. Low levels of physical activity are expected in patients with depression, owing to the reduction of energy levels is one of the hallmarks of the disease. Additionally, daily energy intake was not significantly different between the patients and control group. Lack of significant difference in energy consumption between the two groups was probably due to the fact that patients were new cases. In other words, the duration of their disease was short and the disease-related disorders had not affected their food choices yet.

In the current study, as the most important result, the intermediary role of serum folate and vitamin B12 was investigated in the association between dietary patterns and depression. According to our results, the increase of serum folate and vitamin B12 in healthy dietary pattern and the decrease of these two vitamins in unhealthy dietary pattern were related to depression.

Generally, healthy dietary patterns contain more healthy foods such as folate and vitamin B12, which have a preventive effect against depression [28]. In the healthy dietary pattern, fruits and vegetables are considered as healthy foods, which contain more folate, compared to unhealthy dietary pattern with low consumption of fruits and vegetables. It is important to note that consumption of several food groups that form a dietary pattern does not mean absence of other food groups in that pattern. In other words, other food groups may also have a limited contribution in a food pattern. For example, while meat is not necessarily included as a specific item in healthy dietary patterns, it never implies complete lack of meat intake. In fact, it means that meat consumption is lower than other food group. In the case of vitamin B12, the human body only needs very low amount of this vitamin, which might be met by low consumption of meat in healthy dietary patterns. Besides, the healthy dietary pattern includes milk as a good source of vitamin B12.

Moreover, the interaction between several foods could intensify the protective effect of folate and vitamin B12 against depression. For instance, in addition to folate and vitamin B12, fruits and vegetables contain non-nutrients with beneficial effects on health in healthy dietary patterns. One notable example of non-nutrients are dietary fibers, which improves both the immune and inflammatory functions by affecting the intestinal microbiota. Besides, phytochemicals, consumed along with dietary fibers, can reduce the oxidative stress and eventually inflammation [29]. Impaired immune function and inflammation are also observed in various disorders, including depression. Furthermore, the interaction between different foods of the unhealthy dietary patterns could also weaken the protective role of folate and vitamin B12 against depression. It is important to note that low attendance of unhealthy food items in healthy dietary patterns could also reduce the risk of depression.

The methylene tetra hydro folate reductase (MTHFR) reduces 10-Methylenetetrahydrofolate to 5-methylenetetrahydrofolate, which is the main active circulatory form of folate. Furthermore, 5-Methylenetetrahydrofolate has a key role in one-carbon metabolism and is essential for methylation of homocysteine to methionine. Methionine is the prerequisite of S-adenosylmethionine and is considered as a common methyl group donor in various methylation reactions in the brain for the synthesis of neurotransmitters such as dopamine, serotonin, and noradrenalin; all play an important role in the etiology of depression [30, 31].

In contrast to the previous results [16, 32–34], we observed no significant difference in the mean serum level of tryptophan and Trp/CAA ratio between the case and control groups, before and after adjustment for the confounding factors. Tryptophan hydroxylase is an enzyme responsible to control the serotonin synthesis. In normal condition, only half of this enzyme is saturated with tryptophan. Therefore, alteration of the tryptophan concentration in blood changes the enzyme saturation and eventually the extent of serotonin synthesis. Since valine, leucine, tyrosine, phenylalanine, and isoleucine compete with tryptophan to pass the blood brain barrier, the tryptophan/competing amino acids (Trp/CAA) ratio should rise to access the brain with more tryptophan [35]. The participants of previous investigations were all either under antidepressant treatment or resistant to the treatment. However, patients of the current study were all new cases of depression with no intake of anti-depressant drugs. Therefore, it might be assumed that at the beginning of the disease, the patient’s body might prevent reduced brain access to tryptophan through a compensatory mechanism. Joyce et al. compared patients with a different genetic polymorphism in the promoter region of the serotonin transporter and other depressed patients. These investigators noted that the first group had higher Trp/CAA ratio and poorer response to serotonergic antidepressant drugs than the second group [36]. Thus, changes in the Trp/CAA ratio among our depressed patients might stem from the polymorphism in their serotonin transporter gene. Due to the lack of the molecular analysis, the possible relationship of genetic polymorphisms in depression-related genes with clinical status or biochemical parameters was not discussed in the current study.

Findings show that the patients were less educated than healthy people. It is documented that high *educated people* are more probably to choose *healthier* food, and have *more* health checks and better health outcomes [37, 38]. This issue can also be seen in high-risk groups such as pregnant mothers or immigrants who are more at risk for depression [6].

Considering the study limitations, the following two factors can be mentioned. Similar to all case-control studies, the temporal relationship between depression and dietary patterns was not realized. Furthermore, due to the financial restrictions biochemical measurements were not conducted for all participants.

To decrease recall bias, which is a limitation of case-control studies, we selected new cases among depressed patients and to minimize selection bias, another limitation of case- control studies, we selected control group from the area in which patient individuals were inhabited. To distribute confounders in two groups homogenously in addition to age and gender the participants were matched according to socio-economic status, as well.

As a case-control study, our information can help individuals to understand the differences of dietary patterns between patients with depression and healthy individuals. We assessed the relationship between dietary patterns and depression before and after adjusting for many confounders and depression risk factors. All adjusted confounders were similar to the confounders of other studies [39–42], except for the dietary pattern, which was adjusted in just one study [9]. In one investigation on non-new cases of depression, the effect of anti-depression drugs was adjusted as a confounder [43]. Nevertheless, the patients of our study were all new cases with no drug use history so we controlled this confounder statistically.

## Conclusion

The healthy dietary pattern is related to the risk of depression via increasing the serum level of folate and vitamin B12. However, the unhealthy dietary pattern is related to the risk of depression via decreasing the serum level of the vitamins. Further studies are suggested to confirm the findings.

## Data Availability

The dataset supporting the conclusions of this article can be made available upon request after approval by the authors.

## Abbreviations

BDI: Beck depression inventory questionnaire
BMI: Body mass index
CI: Confidence interval
DSM: Diagnostic and statistical manual of mental disorders
FFQ: Food frequency questionnaire
HIV: Human immunodeficiency virus
HPLC: High-performance liquid chromatography
MDD: Major depressive disorders
MTHFR: Methylene tetra hydro folate reductase
OR: Odd ratio
SPSS: Statistical package for the social sciences
tHcy: Total homocysteine
Trp: Tryptophan
Trp/CAA: Tryptophan/competing amino acids
